# Preliminary multiplex microarray IgG immunoassay for the diagnosis of toxoplasmosis and rubella

**DOI:** 10.1590/0074-02760160509

**Published:** 2017-06

**Authors:** Priscila T Baschirotto, Marco A Krieger, Leonardo Foti

**Affiliations:** 1 Fundação Oswaldo Cruz-Fiocruz, Instituto Carlos Chagas, Curitiba, PR, Brasil; 2 Instituto de Biologia Molecular do Paraná, Curitiba, PR, Brasil

**Keywords:** toxoplasmosis, rubella, diagnosis, multiplex, liquid microarray

## Abstract

**BACKGROUND:**

During pregnancy, toxoplasmosis and rubella can cause serious damage to the mother and the foetus through vertical transmission. Early diagnosis enables implementation of health measures aimed at preventing vertical transmission and minimising damage caused by these diseases.

**OBJECTIVE:**

Here, we report the development of a multiplex assay for simultaneous detection of IgG antibodies produced during toxoplasmosis and rubella infection.

**METHODS:**

This assay is based on xMap technology. Initially, by singleplex assays, we evaluated the following antigens: one *Toxoplasma gondii* lysate; two antigenic extracts of *T. gondii* (TOX8131 and TOX8122); fragments of *T. gondii* antigens [SAG-1 (amino acids 45-198), GRA-7 (24-100), GRA-1 (57-149), ROP-4, and MIC-3 (234-306)]; two chimeric antigens composed of fragments of SAG-1, GRA-7, and P35 (CTOX and CTOXH); and fragments of *Rubella virus* antigens [E-1 (157-176, 213-239, 374-390), E-2 (31-105), and C (1-123)].

**FINDINGS:**

A multiplex assay to simultaneously diagnose toxoplasmosis and rubella was designed with the best-performing antigens in singleplex and multiplex assays, which included CTOXH, *T. gondii* lysate, TOX8131, E-1, and E-2. The multiplex assay showed 100% sensitivity and specificity for anti-*T. gondii* IgG detection and 95.6% sensitivity and 100% specificity for anti-*R. virus* IgG detection.

**MAIN CONCLUSIONS:**

We found that, despite the difficulties related to developing multiplex systems, different types of antigens (extracts and recombinant proteins) can be used to develop high-performance diagnostic tests. The assay developed is suitable to screen for prior *T. gondii* and *R. virus* infections, because it is a rapid, high-throughput, low-cost alternative to the current standard diagnostic tools, which require multiple individual tests.

Toxoplasmosis and rubella are caused by *Toxoplasma gondii* and *Rubella virus*, respectively, which have been reported worldwide. Primary infections in pregnant women may result in vertical transmission of the pathogens, which can cause congenital disease that significantly affects foetal development. The possible effects include structural and functional sequelae in several systems and organs, miscarriage, foetal deaths, stillbirths, premature births, and development of disease postnatally (Havelaar et al. 2007, Simons et al. 2016).

Since clinical manifestations of toxoplasmosis and rubella can be absent or nonspecific, serological screening for toxoplasmosis and rubella in pregnant women is routinely performed worldwide (Montoya & Rosso 2005, Wandinger et al. 2011). An individual is considered susceptible to infection when specific antibodies are absent from serum. In this case, measures to prevent infection by *T. gondii* or *R. virus* can be implemented to reduce cases of vertical transmission. Detection of specific IgG without IgM antibodies is the classical serological pattern indicating past infection and/or vaccination in the case of rubella (Montoya & Rosso 2005, Vauloup-Fellous & Grangeot-Keros 2007, Montoya & Remington 2008). In most cases, pregnant women are considered immunologically protected, and new events are extremely rare (Banatvala & Brown 2004, Montoya & Rosso 2005). Primary infection during pregnancy is most commonly identified by the detection of IgM and/or IgG. However, because of variations in antibody production following infection, a definitive diagnosis is only possible after additional assessments such as confirmation of a significant increase in antibody titre or seroconversion and IgG avidity testing (Wandinger et al. 2011, Hotop et al. 2012). If acute toxoplasmosis and rubella are confirmed, pregnancy can be terminated based on maternal and/or foetal risks and the law(s) of the country (Banatvala & Brown 2004, Montoya & Remington 2008), or toxoplasmosis therapy can be initiated to reduce foetal and neonatal damage or prevent vertical transmission of *T. gondii* (Montoya & Remington 2008, Villard et al. 2013).

Currently, tests such as enzyme immunoassay (EIA), enzyme-linked fluorescent assay (ELFA), chemiluminescent microparticle immunoassay, and electrochemiluminescence immunoassay are widely applied in clinical laboratories to diagnose toxoplasmosis (Murat et al. 2012, Villard et al. 2013) and rubella (Dimech et al. 2008, Enders et al. 2013). Although sensitive and specific, these techniques have certain limitations, including the large sample volumes required, individual assays needed for each marker investigated, and relatively low throughput because of time-consuming procedures. These limitations can be overcome by multiplex technologies that are amenable to automation. xMap technology involves multiplex testing using a liquid microarray. Beads serve as a solid support and contain differing proportions of red and near-infrared fluorophores that result in different colour codes. Each class of beads can be coupled to a specific capture molecule and used in a multiplex format to detect multiple targets. The microspheres are analysed in a Luminex 100/200 System, which has a laser that excites the R-phycoerythrin conjugates and quantifies antigen-antibody interactions, and another laser that excites the fluorochromes in the microsphere to identify bead colour codes (Kellar & Iannone 2002, Elshal & McCoy 2006).

Most commercial tests incorporate lysates or extracts of *T. gondii* or *R. virus* as specific antibody capture molecules. However, there are technical limitations to their production, such as the need to maintain living parasites and difficulties in the standardisation of cultures (Schmidt et al. 1996, Pfrepper et al. 2005, Holec-Gasior 2013). For these reasons, there are several reports of evaluations of recombinant proteins by EIAs that were designed to replace extracts and lysates of *T. gondii* (Holec-Gasior 2013) and *R. virus* (Schmidt et al. 1996, Liu et al. 2013) used in current tests.

In this study, we developed a single bead-based immunoassay to detect IgG antibodies produced in response to *T. gondii* and/or *R. virus* infection. We showed that this preliminary multiplex platform can be used to develop more informative and rapid tests, and that, despite the difficulties associated with the development of multiplex systems, different types of recombinant proteins, lysates, and extracts can be exploited in multiplex format to produce high-performance diagnostic tests.

## MATERIALS AND METHODS

*Samples* - To develop assays for the diagnosis of toxoplasmosis, we used a quality control panel from the Central Laboratory of Paraná (PR-LACEN). This panel contains 92 anti-*T. gondii* IgG-positive serum samples and 30 negative serum samples. The samples were tested for *T. gondii* IgG by an ELFA assay (Vidas, BioMérieux). There is no clinical data associated with these samples. The presence of IgM and IgG anti-*T. gondii* antibodies, as well as IgG avidities, were determined by the ELFA assays VIDAS Toxo IgM, VIDAS Toxo IgG, and VIDAS Toxo IgG Avidity (BioMérieux), respectively. According to the manufacturer, high-avidity test results are obtained only from individuals who have been infected for at least four months. The panel of samples included those with variable IgG test results, including 42 samples that were weakly positive for IgG (up to 500 IU/mL), 42 moderately positive samples (up to 3000 IU/mL), and 10 strongly positive samples (up to 10,000 IU/mL), with different patterns of avidity (low, medium, and high), and positive or negative results for anti-*T. gondii* IgM antibodies.

To develop assays for the detection of rubella, 23 serum or plasma samples classified as positive for anti-*R. virus* IgG antibodies and two samples negative for anti-*R. virus* IgG antibodies were used. These samples were purchased from SeraCare Life Sciences (Catalogue No. PTR 201). According to the supplier, the samples were evaluated for the presence of anti-*R. virus* IgG and IgM by EIA (Abbott EIA-Rubella IgG, BioWhittaker EIA-Rubella IgG, Abbott EIA-Rubella IgM, and BioWhittaker-Rubella IgM EIA), MEIA (Abbott IMX-Rubella IgG), and latex-agglutination assays (Becton Dickinson Rubella-Total Latex Agglutination, Murex Rubella-Total Latex Agglutination, and Seradyn Rubella-Total Latex Agglutination). There is no clinical information associated with these samples. This performance panel included samples with variable IgG reactivities, IgM anti-*T. gondii*-positive and -negative samples, and three samples from individuals who had recently been immunised.

*Antigens* - To detect anti-*T. gondii* antibodies, we purchased recombinant antigens from Prospec, Inc. (Rehovot, Israel), including SAG-1 [amino acids (aas) 45-198, Catalogue No. TOX-261], GRA-7 (aas 24-100, Catalogue No. TOX-262), GRA-1 (aas 57-149, Catalogue No. TOX-263), ROP-4 (Catalogue No. TOX-266), MIC-3 (aas 234-307, Catalogue No. TOX-264), as well as two chimeric antigens from Fapon Biotech, Inc., that are referred to in this report as CTOX (Catalogue No. GECTOXI101) and CTOXH (Catalogue No. GEETOXC101). These chimeric antigens are composed of epitopes of P29+P30+P35 fragments from *T. gondii* and differ only in that horseradish peroxidase (HRP) is conjugated to the CTOXH antigen. Lysates from whole tachyzoites (RH strain) were obtained from Fitzgerald Industries (Catalogue No. 30-AT56). In addition, two extracts from tachyzoites (RH strain) were obtained from Meridian Life Sciences, Inc. (Catalogue Nos. 8122 and 8131) and are referred to in this report as TOX8122 and TOX8131, respectively.

Recombinant *R. virus* E-1 antigens (aas 157-176, 213-239, and 374-390, Catalogue No. RUB-291), E-2 (aas 31-105, Catalogue No. RUB-292), and core antigen C (aas 1-123, Catalogue No. RUB-293) were purchased from Prospec.

*Coupling of beads to antigens* - Briefly, 10^6^ carboxylated paramagnetic beads (Luminex Corp., TX) were vortexed and mixed by ultrasound bath in three alternating on-off cycles of 30 s. The bead suspension was then washed twice with double-distilled water (ddH_2_O) and suspended in 80 µL of activation buffer (100 mM sodium phosphate, pH 6.2). Solutions (10 µL each) of *N*-hydroxysulfosuccinimide (sulfo-NHS, Pierce, IL) and 1-ethyl-3-(3-dimethylaminopropyl)-carbodiimide hydrochloride (EDC, Pierce, IL), both diluted to 50 mg/mL in ddH_2_O, were added to stabilise the reaction and activate the beads. After mixing, the beads were incubated for 20 min in the dark at 25ºC. The activated beads were subsequently washed with coupling buffer (0.1 M NaHCO_3_, pH 8.0), after which 100 µL of antigen solution was added, and the bead-antigen solution was incubated with shaking at 300 rpm for 2 h. After incubation, the beads were washed in phosphate-buffered saline (PBS) at pH 7.2, 1% bovine serum albumin (BSA), 0.02% Tween 20, and 0.05% sodium azide and suspended in 200 µL of blocking/storage buffer (PBS pH 7.2, 1% BSA, 0.02% Tween 20, and 0.05% sodium azide). The beads were counted using a Beckman Coulter Z2 cell counter and stored at 4ºC protected from light.

*Bead-based immunoassay standard protocol* - Serum samples were diluted 1:100 in assay buffer (PBS at pH 7.2, 1% BSA, 0.02% Tween 20, and 0.05% sodium azide), and 50 µL of the resulting mixture was added to each well. The microspheres were diluted to a concentration of 50 beads/µL in assay buffer containing *Escherichia coli* extract at 0.2 mg/mL, and 50 µL was added to each well of a 96-well plate, resulting in a 1:200 sample dilution. In the singleplex assays, only coupled beads with a single colour code were diluted to 50 beads/µL and added to each well in a total of 2500 beads/well. In the multiplex assays, a single dilution of coupled microspheres containing different colour codes was used, each with a concentration of 50 beads/µL. Thereafter, each well received 2500 beads of each colour code.

Diluted serum (50 µL) and beads (50 µL) were mixed and incubated for 15 min in the dark. The beads were then washed twice with 100 µL of wash buffer (PBS pH 7.2, 1% BSA, 0.02% Tween 20, 0.05% sodium azide), 100 µL of 1:1000-diluted goat anti-human IgG conjugated to R-phycoerythrin (Moss Substrates, MO, GTIG-001) was added, and the beads were incubated for 15 min in the dark. The beads were washed twice with 100 µL of wash buffer, and then reporter bead fluorescence and median fluorescence intensity (MFI) were determined using a Luminex 200 reader (Luminex Corp, TX). All incubations were performed at 37ºC on a microplate shaker (300 rpm), and the wash steps were performed with a Hydroflex plate washer with a magnetic plate support (Tecan, NC). For background fluorescence measurements, 50 µL of blocking buffer was added to at least two wells per plate for incubation with beads.

*MFIs and cutoff determinations* - Each antigen and its associated MFI were identified with a Luminex 200 reader. We considered MFI values as valid when the number of microspheres reached a threshold of 100 beads per well. The MFI values were obtained by subtracting the MFI of each sample from the MFI obtained from the average of the background wells (no serum added). The cutoff value for each antigen was defined by the lowest MFI, which gave the highest sensitivity when the specificity reached 100%. Samples were then classified as “positive” or “negative” according to the cutoff values defined for each specific antigen.

*Statistical analysis* - A performance evaluation of the tests was based on an analysis of receiver operating characteristic (ROC) curves, the area under the ROC curve, scatter plots, specificity and sensitivity values, and respective 95% confidence intervals (CIs). Some assays showed similar sensitivity. In such cases, the selection of the best assay was based on a greater distance between the positive MFI and the cutoff value. The Pearson correlation coefficient (r) was calculated from the test results, using the reference method. The ROC curve, area under the curve (AUC), and Pearson correlation coefficient were calculated using the statistical program Medcalc version 12.1.4. Scatter plots of the results were constructed in GraphPad software version 6.0.

*Evaluation of antigens in singleplex assays* - In this initial stage of testing, each antigen was coupled to a single-code bead and tested in singleplex format. This step was performed to analyse the performance of individual antigens and adjust the buffer and antigen concentrations for optimal coupling. The buffers used were 2-(*N*-morpholino)ethanesulfonic acid (MES; 100 mM), PBS, and sodium bicarbonate (NaHCO_3_). The coupling conditions that showed the most satisfactory results were used for further optimisation in subsequent experiments. The antigens with the best performance were selected for use in the multiplex assays.

*Evaluation of antigens in multiplex assays* - The antigens selected were evaluated in multiplex format tests to verify their collective performance and, subsequently, to adjust the test conditions, if necessary. First, all antigens were separately coupled to beads with different colour codes for each antigen using the best concentration and buffer for coupling, as defined during singleplex assay optimisation. Each antigen and its associated MFI were identified with a Luminex 200 reader. Finally, we selected multiplex test conditions for those antigens that performed well when mixed together and that showed the greatest distance between the MFI of the positive sample and the cutoff value. To calculate the overall sensitivity, samples were considered true positives if they gave a positive result with at least one antigen.

## RESULTS

*Antigen detection in singleplex assays* - First, we evaluated the best buffer and antigen concentrations for bead coupling. Then, the antigens that resulted in the best singleplex assay sensitivity were selected for multiplex assays. Supplementary data, Table I and Supplementary data, Figure show sensitivity and specificity results, respectively, as well as scatter plots for all singleplex assays performed with *T. gondii* and *R. virus* antigens evaluated under different coupling conditions.

**TABLE I:** Results of sensitivity and specificity of the best singleplex, multiplex and TR multiplex assays with *Toxoplasma gondii* and *Rubella virus* antigens coupled to beads under optimised conditions

	Format	Antigens	Individual sensitivity (100% specificity; 95% CI)	AUC (95% CI)	Overall sensitivity^*a*^ (100% specificity)
Rubella IgG detection	Singleplex	E1	78% (56-92.5%)	0.826 (0.6659 - 0.9863)	-
		E2	78% (56-92.5%)	0.7826 (0.6140 - 0.9512)	
		C	57% (34.5-77%)	0.7174 (0.4527 - 0.9821)	

	Optimised Rubplex	E1	91% (72-99%)	0.9674 (0.8908 - 1)	100%
		E2	96% (78-99%)	0.9783 (0.919 - 1)	
		C	35% (16-57%)	0.6304 (0.2135 - 1)	

	TR Multiplex	E1	70% (47-87%)	0.8370 (0.5824 - 1)	95.6%
		E2	96% (78-100%)	0.9783 (0.9190 - 1)	

Toxoplasmosis IgG detection	Singleplex	*T. gondii* lysate	100% (96-100%)	1.0000 (1 - 1)	-
		TOX8131	100% (96-100%)	1.0000 (1 - 1)	
		CTOXH	99% (94-100%)	0.9967 (0.9899 - 1)	
		CTOX	99% (94-100%)	0.9967 (0.9899 - 1)	

	Toxoplex	*T. gondii* lysate	98% (92-100%)	0.9985 (0.9953 - 1)	100%
		TOX8131	96% (89-99%)	0.9981 (0.9943 - 1)	
		CTOXH	99% (94-100%)	0.9960 (0.9878 - 1)	
		CTOX	99% (94-100%)	0.9936 (0.9809 - 1)	

	TR Multiplex	*T. gondii* lysate	98% (92-100%)	0.9959 (0.9885 - 1)	100%
		TOX8131	96% (88-99%)	0.9950 (0.9906 - 1)	
		CTOXH	100% (96-100%)	1.0000 (1 - 1)	

AUC: area under the curve; CI: confidence interval. Fragments composition of recombinant antigens: E-1 [157-176, 213-239, 374-390], E-2 [31-105], and C [1-123], CTOX and CTOXH [P29+P30+P35]. TOX8131 (*T. gondii* extract). The optimised coupling conditions can be verified in Supplementary data, Table I. a: overall sensitivities were calculated only for multiplex assays. To calculate overall sensitivity, true positive samples were considered positive if they gave a positive result for at least one antigen.

After evaluating different coupling conditions, we determined that the SAG-1, GRA-7, GRA-1, and ROP-4 antigens did not perform optimally, and they were not evaluated further. In contrast, assays using *T. gondii* antigens MIC-3 (234-306), *T. gondii* lysate, TOX8122, TOX8131, CTOX, and CTOXH, when coupled optimally, resulted in ~100% specificity and sensitivities of 99% (95% CI: 94-100%), 100% (95% CI: 96-100%), 97% (95% CI: 91-99%), 100% (95% CI: 96-100%), 99% (95% CI: 94-100%), and 99% (95% CI: 94-100%), respectively. Among them, the assays using *T. gondii* antigens TOX8131, *T. gondii* lysate, CTOX, and CTOXH coupled under optimal conditions showed the greatest distance between positive MFIs and the cutoff values; therefore, these antigens were selected for the multiplex assay for toxoplasmosis diagnosis.

The assays for IgG anti-*R. virus* detection using optimally coupled antigens E-1, E-2, and C resulted in a specificity of 100% and sensitivities of 78% (95% CI: 56-92.5%), 78% (95% CI: 56-92.5%), and 57% (95% CI: 34.5-77%), respectively. Singleplex assays with antigens E-1 and E-2 performed best, and at least one of these antigens was successfully detected in all samples that were positive. Our antigen selection protocol proved robust, because the samples that gave a false negative result for one particular antigen were positive with at least one of the other antigens. Hence, the utilisation of all antigens yielded a more reliable diagnostic test because the false negative rate was minimised. For this reason, we decided to evaluate antigens E-1, E-2, and C in multiplex assays for rubella diagnosis.

*Antigen evaluation in multiplex assays* - Antigens previously selected and individually coupled to beads under optimised conditions were evaluated in multiplex format tests to verify and optimise their collective performance related to overall sensitivity, which corresponds to proportion of positive results for true positive samples with at least one antigen.

*Multiplex assay for detecting anti-T. gondii IgG (Toxoplex assay)* - The sensitivity and specificity of the Toxoplex assay in detecting CTOX, CTOXH, *T. gondii* lysate, and TOX8131 are presented in Table I. Comparing Toxoplex and singleplex assay results (Fig. 1) revealed that samples characterised by very low IgG anti-*T. gondii* titers showed a 37% and 51% decrease in MFI for TOX8131 and *T. gondii* lysate, respectively, resulting in some false negative results. For this reason, tests performed with the antigenic extract TOX8131 and *T. gondii* lysate showed lower sensitivity (96%, 95% CI: 89-99%; and 98%, 95% CI: 92-100%, respectively) when compared with the 100% sensitivity obtained when testing in singleplex format. However, CTOX and CTOXH, which showed 99% (95% CI: 94-100%) sensitivity, were particularly useful for detecting samples with low IgG levels. Thus, the combination of TOX8131, *T. gondii* lysate, and CTOXH was selected for the final multiplex assay, because all samples were positive for at least one of these antigens, resulting in a 100% overall sensitivity and specificity. CTOX and CTOXH antigens differ only by the presence of HRP, and CTOXH antigen was selected for multiplexing because of it showed the highest AUC among the respective assays, as shown by Table I.

*Multiplex assay for detecting anti-R. virus IgG (Rubplex assay)* - Antigens E-1, E-2, and C, coupled optimally for singleplex assays, were evaluated in a multiplex assay, referred to here as the Rubplex assay. The Rubplex assay showed an overall sensitivity of 82% and a specificity of 100%. Antigens E-1, E-2, and C showed 100% specificity and individual sensitivities of 53% (95% CI: 34.5-77%), 70% (95% CI: 47-87%), and 30% (95% CI: 13-53%), respectively.

The overall sensitivity of this multiplex assay was not satisfactory; thus, optimisation steps were necessary. Comparing the results of the Rubplex assay with those of the singleplex assays, the observed decrease in sensitivity could have been related to an increased consumption of conjugate, because the multiplex assay employed three times more microspheres than did the singleplex assays (7500 vs. 2500). In addition, the use of a higher concentration of serum might also have increased signal intensity. For these reasons, we evaluated additional serum dilutions and secondary antibodies conjugated with R-phycoerythrin.

Assays were performed with samples diluted 1:200, with the phycoerythrin conjugate diluted from 1:500 to 1:100. Additional assays were performed with samples diluted 1:100, using the phycoerythrin conjugate diluted to 1:1000, 1:500, or 1:100.

As shown in Supplementary data, Table II, test conditions in which the serum and phycoerythrin conjugate were diluted to 1:100 showed the highest overall sensitivity and specificity (equivalent to 100%). Antigens E-1, E-2, and C showed 100% specificity and individual sensitivities of 91% (95% CI: 72-99%), 96% (95% CI: 78-99%), 35% (95% CI: 16-57%), respectively. The reduced sensitivity and MFI of positive samples revealed that the multiplex assay was still not optimised when compared to the singleplex assays, which was clearly a result of insufficient quantities of sample and phycoerythrin conjugates. Further optimisation resulted in a general increase in the MFIs and better separation from the cutoff value, as shown in Figure. Antigen C underperformed and, thus, was not selected for use in the Toxo+Rub (TR) multiplex assay.

**TABLE II:** Pearson’s correlation between enzyme-linked fluorescent assay (ELFA), enzyme immunoassay (EIA) and multiplex enzyme immunoassay (MEIA) assays, and the best singleplex, multiplex and TR multiplex assays using *Rubella virus* and *Toxoplasma gondii* antigens coupled to beads under optimised conditions

	Format	Antigens	Pearson’s correlation		

			Abbott IgG, EIA	Abbott IMX IgG, EIA	BioWhittaker IgG, EIA
Rubella IgG detection	Singleplex	E1	(+) 0.4	(+) 0.31	(+) 0.47
		E2	(-) 0.19	(-) 0.36	(-) 0.23
		C	(+) 0.27	(+) 0.18	(+) 0.24

	Optimised Rubplex	E1	(+) 0.25	(+) 0.03	(+) 0.44
		E2	(-) 0.02	(-) 0.17	(-) 0.11
		C	(+) 0.31	(+) 0.22	(+) 0.17

	TR Multiplex	E1	(+) 0.23	(-) 0.09	(+) 0.3
		E2	(-) 0.05	(-) 0.25	(-) 0.24

			VIDAS IgG bioMérrieux, ELFA		
Toxoplasmosis IgG detection	Singleplex	*T. gondii* lysate	(+) 0.41		
		TOX8131	(+) 0.45		
		CTOXH	(+) 0.08		
		CTOX	(+) 0.15		

	Toxoplex	*T. gondii* lysate	(+) 0.43		
		TOX8131	(+) 0.47		
		CTOXH	(+) 0.34		
		CTOX	(+) 0.26		

	TR Multiplex	*T. gondii* lysate	(+) 0.30		
		TOX8131	(+) 0.31		
		CTOXH	(+) 0.17		

A Pearson’s coefficient value between 0 and 0.3 indicated a weak correlation; between 0.3 and 0.7, a moderate correlation; and above 0.7, a strong correlation. The effect of the correlation was determined by the positive or negative sign of the coefficient. Fragments composition of recombinant antigens: E-1 [157-176, 213-239, 374-390], E-2 [31-105], and C [1-123], CTOX and CTOXH [P29+P30+P35]. TOX8131 (*T. gondii* extract). The optimised coupling conditions can be verified in Supplementary data, Table I.

*Multiplex assay for the detection of anti-T. gondii and anti-R. virus IgGs (TR multiplex assay)* - The TR multiplex assay, including the antigens TOX8131, *T. gondii* lysate, CTOXH, E-1, and E-2, was performed using serum and secondary antibody dilutions of 1:100, which were ideal test conditions for the Rubplex assay. The overall sensitivity and specificity of each antigen in the TR multiplex assay compared to those of the singleplex, Toxoplex, and Rubplex assays are shown in Table I.

For toxoplasmosis diagnosis, the TR multiplex showed 100% overall sensitivity and specificity, because all true positive samples were detected by at least one of the *T. gondii* antigens selected for the tests. For rubella diagnosis, the TR multiplex showed 95.6% overall sensitivity and 100% specificity. Figure shows a comparison of the best singleplex and multiplex assays for each of the antigens tested, revealing the impact of multiplexing and changing the assay parameters on the MFI. The use of a lower dilution of both the serum and phycoerythrin conjugate reduced the sensitivity of detecting *T. gondii* lysate and TOX8131 antigens by specific antibodies.

Table II shows the Pearson’s correlation coefficients for each antigen in the singleplex, Rubplex, and TR multiplex formats with respect to results obtained from tests used to characterise the serological panel.

The ELFA method and liquid microarray assays using *T. gondii* lysate and TOX8131 showed a moderate positive correlation between all test formats. In general, the chimeric antigens CTOX and CTOXH showed a less robust positive correlation.

Correlations between the singleplex and multiplex assay results based on EIAs and MEIAs using *R. virus* antigens varied considerably. In general, the results of tests performed with E-1 correlated strongly among these methods, whereas those obtained with E-2 were poorly correlated.

## DISCUSSION

Here, we developed a preliminary assay based on liquid microarray technology and evaluated 13 antigens for the detection of IgG anti-*T. gondii* or anti-*R. virus*. The performance of six of these antigens was sufficiently robust for their inclusion in a multiplex diagnostic test for rubella and toxoplasmosis. This multiplex assay showed 100% overall sensitivity and specificity for toxoplasmosis detection, and 95.6% overall sensitivity and 100% specificity for rubella detection. In addition, we showed that E1, E2, and chimeric CTOXH (P29+P30+P35) recombinant proteins provided satisfactory results, so that their application in immunoassays may be further optimised.

The protein concentration and pH of the buffer used for sample dilution are critical factors that influence the quality of bead coupling and, hence, the quality of the assay. The bonds between the carboxyl groups of microspheres and free amino groups present in the antigens are mainly ionic, and are thus controlled by the pH of the buffer used. The buffer pH can cause conformational changes in the structure of the molecule, thereby altering the sites of interaction with specific antibodies. The optimal antigen concentration in a microsphere depends on the antibody titers in the blood, as well as the dilution of the plasma or serum used in the assay. In the current study, this optimisation process increased the sensitivity of detection for E-1 (from 57% to 82%), E-2 (from 43% to 78%), C (from 9% to 57%), and MIC-3 (from 66% to 99%).

The performance of a test is affected by the choice of samples that are included in the test panel. The panel of samples used for the validation tests to diagnose rubella comprised only two negative samples. Obtaining negative samples is currently hampered by the extensive vaccination campaign being conducted against rubella in Brazil, which has almost eradicated the disease in the country. However, the panel of toxoplasmosis and rubella samples are representative of the various stages of infection, including seroconversion and acute, chronic, and late stage samples. Moreover, 40% of positive samples in both panels showed low reactivity for specific IgG in gold standard assays, yielding unbiased sensitivity results. Importantly, there were no discordant results. The assays developed in this work serve as proof of concept. Validation tests must be conducted, including tests with a number of representative samples from the population to be studied and based on the acceptable margin of error and other validation criteria such as the detection limit, linearity, absolute recovery, reproducibility, repeatability, and analytical sensitivity.

The singleplex assays performed with TOX8131, TOX8122, and *T. gondii* lysate antigens and CTOXH (P29+P30+P35) showed sensitivities of 100%, 97%, 100%, and 99%, respectively, and a specificity of 100% in each case. This excellent performance was related to the fact that the antigenic extracts and chimeric antigen provided a wider range of epitopes for binding anti-*T. gondii* antibodies compared to those of individual antigens. During infection, a wide variety of antigens are present, as they define exposure to the immune system and determine the avidity of IgG-specific antibodies, and stage of infection. These factors lead to noticeable variation in the intensity of humoral responses elicited against different *T. gondii* antigens (Ferrandiz et al. 2004, Pfrepper et al. 2005, Holec et al. 2008, Holec-Gasior 2013). Thus, the increased variety of epitopes in the *T. gondii* extracts and lysate antigen resulted in the best Pearson’s correlations by ELFA, which employs an antigenic extract as the capture molecule. Likewise, differences in performance between the assays using *T. gondii* extract and lysate were related to differences in antigenic composition.

In singleplex and all multiplex assays, the E-1 and E-2 antigens performed significantly better than the C antigen. Both vaccination and natural infection by *R. virus* elicit a host immune response against the structural proteins E-1, E-2, and C (Chaye et al. 1992a, Vauloup-Fellous & Grangeot-Keros 2007). The mosaic E-1 protein employed in this study includes the amino acid fragment 214-240 containing the hemagglutinin epitope, fragments 214-233 and 219-233, which correspond to neutralising epitopes, and fragment 374-390, all of which are highly recognised in enzyme-linked immunosorbent assays (ELISAs) (Chaye et al. 1992b). The E-2 and C antigens were limited to fragments 31-105 and 1-123, which contain recognised epitopes (Wolinsky et al. 1991, 19, 1993). Antigen E-1 is more immunogenic in adults and children, vaccinated individuals and those with recent infections and who are currently infected, and children with congenital rubella syndrome) (Chaye et al. 1992a, Nedeljkovic et al. 1999, Wilson et al. 2006). This finding explains why E-1 showed the best Pearson’s correlation between all assays, as E-1 was present in the *R. virus* extracts used in tests to characterise the serostatus of the sample panel.

The development of multiplex assays presents many challenges related to the use of different antigens and antibodies in a single assay, as well as the same assay parameters to detect different targets (de Jager et al. 2003, Elshal & McCoy 2006). Here, dilution of serum and secondary antibody to 1:100 was optimal for detecting *R. virus* antigens. However, the use of these dilutions and increased complexity of the multiplex system led to reduced performance with respect to TOX8131 and *T. gondii* lysate, compared to their performances in singleplex assays. Nevertheless, the multiplex assay resulted in 100% detection of IgG anti-*T. gondii* in all positive samples. CTOXH showed 100% sensitivity in the TR multiplex assay, which indicates that including multiple antigens can replace antigenic extracts produced from *T. gondii* cultures, as seen in toxoplasmosis diagnostics (Holec-Gasior 2013). Therefore, a multiplex assay can be developed including only this chimeric antigen for the detection of anti-*T. gondii* IgG antibodies.

Our multiplex assay offers benefits in terms of time, cost, and sample volume required for testing, as previously reported for other multiplex assays when compared to conventional immunoassays (Binnicker et al. 2010, 2011, Dhiman et al. 2010). Researchers using AtheNA Multi-Lyte (Zeus Scientific) and Bioplex (Bio-Rad Laboratories) platforms, which are based on similar principles and exhibit similar performances as the Luminex system, achieved very similar results for anti-*T. gondii* (Binnicker et al. 2010) and anti-*R. virus* IgG antibody detection (Binnicker et al. 2010, 2011, Dhiman et al. 2010). The time from the beginning of the assay to detecting six different targets with the Luminex system in a 96-well format was three hours (including assay reading), which corresponds to approximately 15 min for each molecule employed in antigen capture. Thus, the efficiency and throughput is increased when a greater number of targets is simultaneously detected, particularly when compared to a single ELISA assay that takes 5 h to perform. Reducing the required hands-on time enables operators to perform almost two full Luminex assays in the same time needed to perform a single ELISA.

Furthermore, the cost of reagents for this multiplex assay is 60% lower than the costs for equivalent ELISA assays used to diagnose both diseases, excluding the costs related to laboratory operating times. Moreover, unlike ELISA assays, the cost of our multiplex assay can be further reduced by including additional targets, as this requires only the cost of adding the microspheres and antigens and maintains the same amounts of other reagents and labor.

This multiplex assay for the detection of anti*-T. gondii* and anti-*R. virus* IgGs required the use of only 2 µL of serum or plasma. This is a significant improvement, particularly for use with pregnant women, because current prenatal screening for multiple infectious diseases requires large volumes of blood samples.

In conclusion, the multiplex assay, which includes *R. virus* recombinant antigens and *T. gondii* chimeric antigens, extract, and lysate, showed promising sensitivities and specificities. This multiplex assay for specific IgG detection may be useful for determining whether pregnant woman have had prior contact with the pathogens that cause toxoplasmosis and rubella, as the analysis of anti-*R. virus* IgG antibodies in women already vaccinated is useful for surveillance of immunity against *R. virus*, which allows a public health risk assessment. However, this assay is not sufficient for toxoplasmosis and rubella diagnosis. For this purpose, assays that specifically detect IgM can be integrated or developed on separate platforms for IgG and IgM detection. This decision depends on the assay formats used, because there is the possibility of cross-reaction between test components. Avidity assays can be included as an additional step after the first IgG fluorescence reading. It is important to understand that the specific IgG response may evolve differentially with different antigens, so results positive for one specific antigen and negative for others must be confirmed through an analysis of paired samples, which is already common. The test parameters were calculated based on a well-characterised set of sera, but the number of samples was limited, so the performance of the assay must be further validated with a larger number of samples. The Luminex-based multiplex assay is rapid and can process large numbers of samples, which is clearly advantageous for screening programs in a public health setting. In this way, this multiplex assay shows the potential to be used as a platform for the inclusion of additional markers of prenatal infections for systematic screening of pregnant women.

**Figure:**
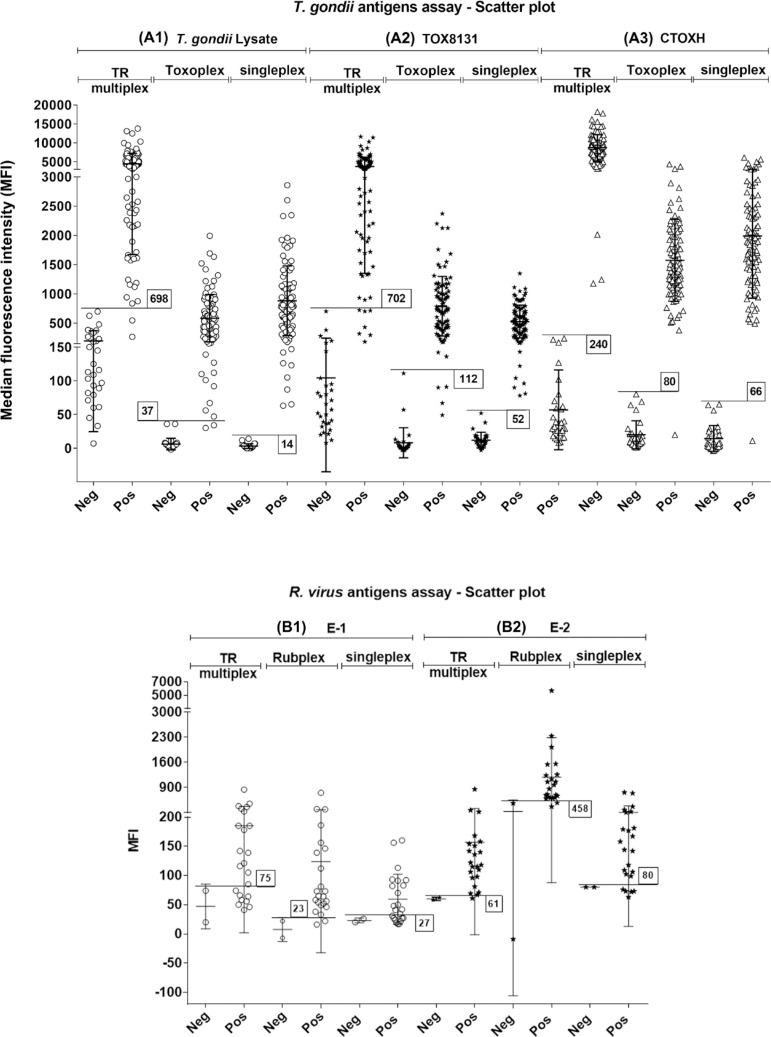
Scatter plot of the best singleplex, multiplex, and TR multiplex assays for specific IgG detection by *Toxoplasma gondii* and *Rubella virus* antigens. Horizontal lines represent cutoff values. MFI: median fluorescence intensity; Neg: negative; Pos: positive. Singleplex antigen were evaluated against the sample panel; in the multiplex assay, all antigens were evaluated together against the sample panel. (A1) Scatter plot results of singleplex, multiplex, and TR multiplex assays for *T. gondii* lysate, which had cutoff values of 14, 37, and 698, respectively. (A2) Scatter plot results of singleplex, multiplex, (Toxoplex) and TR multiplex assays for TOX8131, which had cutoff values of 52, 112, and 702, respectively. (A3) Scatter plot results of singleplex, multiplex (Toxoplex), and TR multiplex assays for CTOXH, which had cutoff values of 66, 80, and 240, respectively. (B1) Scatter plot results of singleplex, multiplex (Rubplex), and TR multiplex assays for E-1, which showed cutoff values of 27, 23, and 75, respectively. (B2) Scatter plot results of singleplex, multiplex (Rubplex), and TR multiplex assays for E-2, which showed cutoff values of 80, 458, and 61, respectively.
