# Supplementary material for: Preliminary multiplex microarray IgG immunoassay for the diagnosis of toxoplasmosis and rubella
Source: Mem Inst Oswaldo Cruz. 2017 Jun;112(6):428–36. doi: 10.1590/0074-02760160509 (PMC5446232; doi:10.1590/0074-02760160509)
Supplement: Supplementary file 1 [file 0074-0276-mioc-112-6-0428-suppl01.pdf]

TABLE I

Results of sensitivity and specificity of singleplex assays for the detection of IgG anti-*Toxoplasma gondii* and *Rubella virus*

T. gondii Ags	Coupling buffer	Antigen concentration (µg/mL)	Sensitivity (specificity 100%)	AUC
SAG-1	PBS (pH 7.2)	50	3%	0.619
GRA-7	PBS (pH 7.2)	100	7%	0.630
GRA-1	MES (pH 5.5)	50	7%	0.757
	MES (pH 5.0)	50	15%	0.751
MIC-3	MES (pH 6.5)	125	86%	0.999
	MES (pH 5.5)	100	87%	0.970
	MES (pH 5.5)	125	66%	0.999
	MES (pH 5.5)	150	85%	0.970
	MES (pH 5.0)	100	93%	0.985
	MES (pH 5.0)	125	99%	0.999
T. gondii lysate	PBS (pH 7.2)	100	100%	1.0
	PBS (pH 7.2)	150	100%	1.0
	PBS (pH 7.2)	200	100%	1.0
	PBS (pH 7.2)	250	100%	1.0
	PBS (pH 7.2)	500	100%	1.0
TOX 8122	PBS (pH 7.2)	100	97%	0.997
	PBS (pH 7.2)	150	97%	0.998
	PBS (pH 7.2)	200	96%	0.997
TOX 8131	PBS (pH 7.2)	100	99%	0.997
	PBS (pH 7.2)	150	100%	1.0
	PBS (pH 7.2)	200	100%	1.0
CTOX	MES (pH 6.5)	100	99%	0.987
	PBS (pH 7.2)	100	99%	0.997
	$NaHCO_3$ (pH 8.0)	100	99%	0.994
CTOXH	MES (pH 6.5)	100	99%	0.995
	PBS (pH 7.2)	100	99%	0.995
	$NaHCO_3$ (pH 8.0)	100	99%	0.996

R. virus Ags	Coupling buffer	Antigen concentration (µg/mL)	Sensitivity (specificity 100%)	AUC
E-1	MES (pH 6.5)	50	70%	0.739
	PBS (pH 7.2)	50	57%	0.663
	$NaHCO_3$ (pH 8.0)	50	70%	0.728
	MES (pH 6.5)	100	61%	0.652
	PBS (pH 7.2)	100	82%	0.870
	$NaHCO_3$ (pH 8.0)	100	78%	0.826
	$NaHCO_3$ (pH 8.0)	150	70%	0.739
E-2	MES (pH 6.5)	50	70%	0.717
	PBS (pH 7.2)	50	57%	0.587
	$NaHCO_3$ (pH 8.0)	50	78%	0.793
	MES (pH 6.5)	100	48%	0.674
	PBS (pH 7.2)	100	61%	0.717
	$NaHCO_3$ (pH 8.0)	100	65%	0.717
	$NaHCO_3$ (pH 8.0)	150	43%	0.717
	$NaHCO_3$ (pH 8.0)	25	78%	0.783
C	MES (pH 6.5)	50	57%	0.717
	PBS (pH 7.2)	50	9%	0.543
	$NaHCO_3$ (pH 8.0)	50	23%	0.533
	MES (pH 6.5)	100	39%	0.609
	PBS (pH 7.2)	100	13%	0.520
	$NaHCO_3$ (pH 8.0)	100	22%	0.533
	MES (pH 6.5)	150	19%	0.707
	MES (pH 6.5)	25	57%	0.583

The best coupling conditions are marked in gray. AUC: area under the curve.

TABLE II

Optimisation of sera and R-phycoerythrin (R-PE) conjugate dilutions in Rubplex assay for specific IgG detection

	Dilution (serum and R-PE conjugate)	Antigen	Coupling buffer	Antigen concentration (μg/mL)	Sensitivity	Specificity	Overall sensitivity and specificity
Multiplex assay	Serum: 1:200 conjugate:1:500	E-1	$NaHCO_3$ (pH 8.0)	100	74%	100%	96-100%
		E-2	$NaHCO_3$ (pH 8.0)	25	65%	100%	
		C	MES (pH 6.5)	50	65%	100%	
	Serum: 1:200 conjugate:1:100	E-1	$NaHCO_3$ (pH 8.0)	100	74%	100%	91-100%
		E-2	$NaHCO_3$ (pH 8.0)	25	61%	100%	
		C	MES (pH 6.5)	50	43%	100%	
	Serum: 1:100 conjugate:1:1000	E-1	$NaHCO_3$ (pH 8.0)	100	48%	100%	87-100%
		E-2	$NaHCO_3$ (pH 8.0)	25	57%	100%	
		C	MES (pH 6.5)	50	45%	100%	
	Serum: 1:100 conjugate:1:500	E-1	$NaHCO_3$ (pH 8.0)	100	76%	100%	91-100%
		E-2	$NaHCO_3$ (pH 8.0)	25	57%	100%	
		C	MES (pH 6.5)	50	30%	100%	
	Serum: 1:100 conjugate:1:100	E-1	$NaHCO_3$ (pH 8.0)	100	91%	100%	100-100%
		E-2	$NaHCO_3$ (pH 8.0)	25	96%	100%	
		C	MES (pH 6.5)	50	35%	100%	

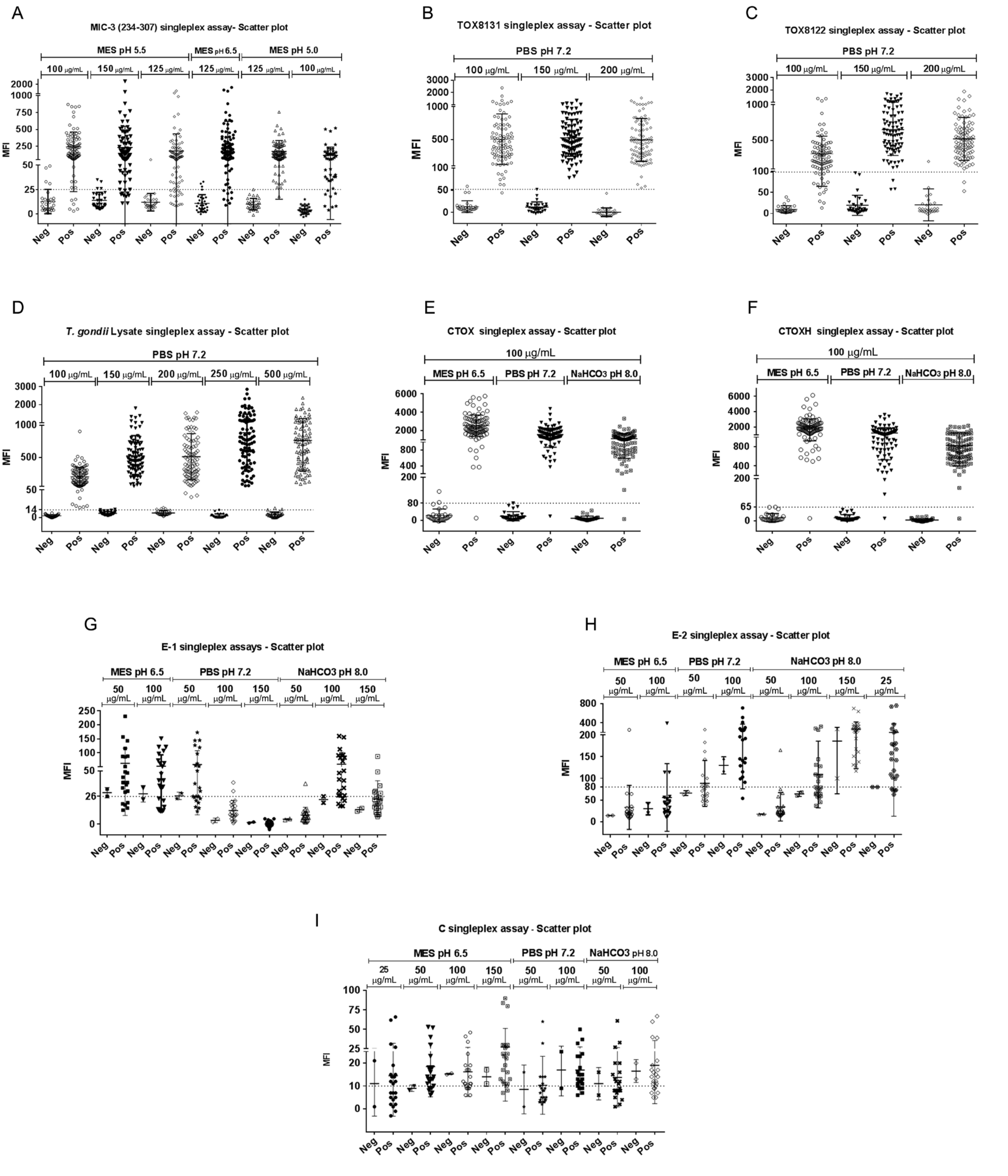

Scatter plots of singleplex assays with *Toxoplasma gondii* and *Rubella virus* antigens. Line represents cutoff value of the best assay. MFI: median of fluorescence intensity; Neg: negative; Pos: positive. (A) Results of MIC-3 antigen coupling in MES buffer, pH 5.5, at 100 µg/mL, 125 µg/mL and 150 µg/mL; MES, pH 6.5, at 125 µg/mL; and MES, pH 5.0, at 100 µg/mL and 125 µg/mL. (B) TOX8131 antigen coupled in PBS buffer, pH 7.2, at 100 µg/mL, 150 µg/mL and 200 µg/mL. (C) TOX8122 antigen coupled in PBS buffer at 100 µg/mL, 150 µg/mL, and 200 µg/mL. (D) Results for the *T. gondii* lysate coupled in PBS buffer, pH 7.2, at 100 µg/mL, 150 µg/mL, 200 µg/mL, 350 µg/mL, and 500 µg/mL. (E) CTOX antigen coupled at 100 µg/mL in MES, pH 6.5, PBS, pH 7.2, and $NaHCO_3$, pH 8.0. (F) CTOXH antigen coupled in 100 µg/mL in MES, pH 6.5, PBS, pH 7.2, and $NaHCO_3$, pH 8.0. (G) E-1 antigen coupled in MES buffer, pH 6.5, at 50 µg/mL and 100 µg/mL; in PBS, pH 7.2, at 50 µg/mL, 100 µg/mL, and 150 µg/mL; and in $NaHCO_3$, pH 8.0, at 50 µg/mL, 100 µg/mL, and 150 µg/mL. (H) E-2 antigen coupled in MES buffer, pH 6.5, at 50 µg/mL and 100 µg/mL; in PBS at 50 µg/mL and 100 µg/mL; and $NaHCO_3$, pH 8.0, at 50 µg/mL, 100 µg/mL, 150 µg/mL, and 25 µg/mL. (I) C antigen coupled in MES buffer, pH 6.5, at 25 µg/mL, 50 µg/mL, 100 µg/mL, and 150 µg/mL, in PBS, pH 7.2, at 50 µg/mL, 100 µg/mL, and in $NaHCO_3$ at 50 µg/mL and 100 µg/mL.
